# Mutation Frequency in Main Susceptibility Genes Among Patients With Head and Neck Paragangliomas

**DOI:** 10.3389/fgene.2020.614908

**Published:** 2020-12-18

**Authors:** Anastasiya V. Snezhkina, Maria S. Fedorova, Vladislav S. Pavlov, Dmitry V. Kalinin, Alexander L. Golovyuk, Elena A. Pudova, Zulfiya G. Guvatova, Nataliya V. Melnikova, Alexey A. Dmitriev, George S. Razmakhaev, Andrey A. Poloznikov, Galina S. Alekseeva, Andrey D. Kaprin, George S. Krasnov, Anna V. Kudryavtseva

**Affiliations:** ^1^Engelhardt Institute of Molecular Biology, Russian Academy of Sciences, Moscow, Russia; ^2^Vishnevsky Institute of Surgery, Ministry of Health of the Russian Federation, Moscow, Russia; ^3^National Medical Research Radiological Center, Ministry of Health of the Russian Federation, Moscow, Russia

**Keywords:** head and neck paragangliomas, carotid and vagal paragangliomas, susceptibility genes, SDHx, mutation frequency, pathogenic mutations

## Abstract

Head and neck paragangliomas (HNPGLs) are rare neuroendocrine tumors that have a high degree of heritability and are predominantly associated with mutations in ten genes, such as *SDHx, SDHAF2, VHL, RET, NF1, TMEM127, MAX, FH, MEN2*, and *SLC25A11*. Elucidating the mutation prevalence is crucial for the development of genetic testing. In this study, we identified pathogenic/likely pathogenic variants in the main susceptibility genes in 102 Russian patients with HNPGLs (82 carotid and 23 vagal paragangliomas) using whole exome sequencing. Pathogenic/likely pathogenic variants were detected in 43% (44/102) of patients. We identified the following variant distribution of the tested genes: *SDHA* (1%), *SDHB* (10%), *SDHC* (5%), *SDHD* (24.5%), and *RET* (5%). *SDHD* variants were observed in the majority of the patients with bilateral/multiple paragangliomas. Thus, among Russian patients with HNPGLs the most frequently mutated gene was *SDHD* followed by *SDHB, SDHC, RET*, and *SDHA*.

## Introduction

Head and neck (HN) paragangliomas (PGLs) are rare neuroendocrine tumors of four distinct localizations: carotid, vagal, laryngeal, and middle ear PGLs. Carotid paragangliomas (CPGLs) arise from the carotid glomus at the carotid artery bifurcation and are the most common form of HNPGLs (60%) (El-Naggar et al., 2017). Middle ear and vagal paragangliomas (MEPGLs and VPGLs) are less frequent than CPGLs and account for 29% and 13%, respectively; laryngeal PGLs are very rare (El-Naggar et al., 2017). All the HNPGLs are often characterized by slow growth and non-aggressive behavior, but exhibit metastatic potential. The overall metastatic rate for HNPGLs vary depending on the site of tumor localization: 2% for larynx and middle ear, 4–6% for carotid, and up to 16% for vagal PGLs (Williams, 2017). HNPGLs can also develop as bilateral or multiple tumors and pose significant treatment challenges.

HNPGLs can as familial or sporadic forms (Dahia, 2014). Familial HNPGLs together with pheochromocytomas (PCCs) account for about 40% and are associated with four types of hereditary paraganglioma syndromes (PGL1–5) caused by mutations in the following genes: *SDHD* (PGL1), *SDHAF2* (PGL2), *SDHC* (PGL3), *SDHB* (PGL4), and *SDHA* (PGL5) (Burnichon et al., 2010; Boedeker et al., 2014). Patients with hereditary HNPGLs less frequently harbor germline mutations in *TMEM127* (Bausch et al., 2017), *RET* (Kudryavtseva et al., 2019), *MAX* (Burnichon et al., 2012), *FH* (Castro-Vega et al., 2014), and *SLC25A11* (Buffet et al., 2018). Germline mutations in *VHL, NF1*, and *MEN2* have been detected in HNPGLs in association with other tumoral and clinical features (Boedeker et al., 2009).

In this study, we aimed to assess the frequency of variants in the main susceptibility genes for HNPGLs, such as *SDHx, SDHAF2, VHL, RET, NF1, TMEM127, MAX, FH, MEN2*, and *SLC25A11*, among a representative set of Russian patients with CPGLs and VPGLs.

## Materials and Methods

### Patients

In total, 102 Russian patients with HNPGLs, including 82 patients with CPGLs and 23 with VPGLs, were enrolled at the Vishnevsky Institute of Surgery, Ministry of Health of the Russian Federation. Informed consent was obtained from all patients. This study was approved by the Ethics Committee of Vishnevsky Institute of Surgery with ethics committee approval no. 004-2020, 03.07.2020, and was performed in accordance with the Declaration of Helsinki (1964) (World Medical Association, 2001). The clinicopathologic characteristics of the patients are presented in Table 1.

**Table 1:** Clinicopathologic characteristics of patients with HNPGLs.

**Characteristic**	**Number of patients, *n* (%)**
Total patients	102
**Tumor localization**	
CPGLs	82 (80%)
VPGLs	23 (22.5%)
**Sex**	
Male	25 (24.5%)
Female	77 (75.5%)
**Age at diagnosis**	
≥40	71 (70%)
<40	31 (30%)
Mean	48 (16–79)
**Tumor characteristics**	
Single	96 (94%)
Bilateral/multiple	6 (6%)
Recurrent	8 (8%)
Metastasis	1 (1%)

### Exome Library Preparation and Sequencing

DNA was extracted from formaldehyde fixed-paraffin embedded (FFPE) tumor tissues using High Pure FFPET DNA Isolation Kit (Roche, Basel, Switzerland) and quantified with a Qubit 2.0 Fluorometer (Thermo Fisher Scientific, Waltham, MA, USA). DNA quality was assessed by quantitative PCR (qPCR) using QuantumDNA Kit (Evrogen, Moscow, Russia). Exome libraries were prepared from DNA using Rapid Capture Exome Kit (Illumina, San Diego, CA, USA) or TruSeq Exome Library Prep Kit (Illumina) according to the manufacturer's recommendations. High-throughput sequencing of the exome libraries was performed on a NextSeq 500 System (Illumina) in a paired-end mode of 76 × 2 bp. The average coverage for each sample was at least 300X. Sequencing data are available at NCBI Sequence Read Archive (SRA) BioProject PRJNA639937.

### Mutation Analysis

Raw sequencing read quality was assessed using FastQC (v. 0.11.9). The reads were trimmed for quality (less than Q20), and adapter sequences were removed using Trimmomatic (v. 0.39) (Bolger et al., 2014). Alignment of reads to the reference human genome GRCh37.75/hg19 was performed with Burrows-Wheeler Aligner (v. 0.7.17) (Li and Durbin, 2010). To report alignment statistics and determine read duplicates, we applied SAMtools (v. 1.10) (Li et al., 2009) and Picard-tools (v. 2.23.4). Base quality score recalibration was carried out with GATK4 (v. 4.1.2) (McKenna et al., 2010) and dbSNP (common variants 2015-06-05) (Sherry et al., 2001). Variant calling was performed with GATK HaplotypeCaller (McKenna et al., 2010). We excluded false positives using StrandBiasBySample, StrandOddsRatio, and BaseQualityRankSumTest annotations, as well as mis-sequenced single-nucleotide variants in polyN motifs, such as GGGTG > GGGGG, CCCCG > CCCCC, and others. For functional annotation of variants, ANNOVAR (v. 20200316) (Wang et al., 2010) was used. Annotations included allele frequency data [gnomAD (Karczewski et al., 2020), Kaviar (Glusman et al., 2011), and ESP-6500 (http://evs.gs.washington.edu/EVS/)], information about reported genomic variations and its association with human pathologies [ClinVar (Landrum et al., 2018), dbSNP, and COSMIC (Tate et al., 2019)], score for the conservation of mutated sites [PhastCons (Siepel et al., 2005) and PhyloP (both PHAST v. 1.5) (Pollard et al., 2010)], localization of variants in protein domains [InterPro (v. 81.0) (Mitchell et al., 2019)], as well as pathogenicity prediction score [SIFT (v. 6.2.1) (Vaser et al., 2016), PolyPhen2 (v. 2.2.2) (Adzhubei et al., 2010), MutationTaster (v. 2013-03-20) (Schwarz et al., 2014), LRT (v. 0.2) (Chun and Fay, 2009), PROVEAN (Choi and Chan, 2015), MetaSVM and MetaLR (Dong et al., 2015), CADD (v. 1.6) (Kircher et al., 2014), and DANN (Quang et al., 2015)].

### Sanger Sequencing

To validate the whole exome sequencing data, Sanger sequencing was performed in Evrogen. Primer sequences are available on request.

## Results

A representative set of HNPGL samples were collected from 102 Russian patients diagnosed with CPGLs (*n* = 82) and VPGLs (*n* = 23), including 76 patients with single CPGL, 20 patients with single VPGL, and 6 patients with bilateral/multiple PGLs (three of them had both carotid and vagal paragangliomas) (Table 1). These tumor samples were analyzed for the presence of pathogenic/likely pathogenic variants in the main susceptibility genes for HNPGLs: *SDHx, SDHAF2, VHL, RET, NF1, TMEM127, MAX, FH, MEN2*, and *SLC25A11*. Variants were classified as pathogenic or likely pathogenic according to the annotations in the ClinVar database or by using the criteria of the American College of Medical Genetics and Genomics and the Association for Molecular Pathology (ACMG-AMP) (Richards et al., 2015).

Pathogenic/likely pathogenic variants were revealed in 44 out of 102 (43%) patients with HNPGLs (Supplementary Table 1). The prevalence of variants was as follows: *SDHA* (1%, 1/102), *SDHB* (10%, 10/102), *SDHC* (5%, 5/102), *SDHD* (24.5%, 25/102), and *RET* (5%, 5/102). Almost all patients with bilateral/multiple paragangliomas (except a patient with no variants in any of the main susceptibility genes tested) demonstrated pathogenic variants in *SDHD* (Table 2). A pathogenic variant NM_003002: c.A305G, p.H102R (chr11: 111959726, rs104894302) was the most frequent *SDHD* mutation detected among the Russian patients. This variant has been found in nine patients with CPGLs and three with VPGLs, including two patients with bilateral/multiple paragangliomas (one patient among them had both tumors subjected to genetic testing). In addition, it is noteworthy that only one variant NM_020975: c.A2372T, p.Y791F (chr10: 43613908, rs77724903) was determined among all *RET*-mutated HNPGLs indicating its high frequency in this population.

**Table 2:** Types of HNPGLs and number of patients with identified pathogenic/likely pathogenic variants.

**Tumor type**	**Total patients, *n***	**Patients with variants, *n***	**Gene variants, *n***				
			***SDHA***	***SDHB***	***SDHC***	***SDHD***	***RET***
CPGLs	82	38	1	7	5	23	4
VPGLs	23	9	0	3	0	5	1
Bilateral/multiple PGLs	6	5	0	0	0	5	0

Also, we analyzed the frequency of variants in the main susceptibility genes separately for CPGLs and VPGLs. In CPGLs, pathogenic/likely pathogenic variants were detected in 38 of 82 (46%) patients and were distributed as follows: *SDHA* (1%, 1/82), *SDHB* (8.5%, 7/82), *SDHC* (6%, 5/82), *SDHD* (28% 23/82), and *RET* (5%, 4/82) (Supplementary Table 1, Table 2). The majority of patients had pathogenic/likely pathogenic variants in one of these genes, however, in two patients, a likely pathogenic variant NM_020975: c.A2372T, p.Y791F (chr10: 43613908, rs77724903) in *RET* were corepresented with *SDHA* (Pat16) and *SDHB* (Pat142) variants. The same variant in *RET* was identified in two other patients (Pat35 and Pat155) with CPGLs. A likely pathogenic variant NM_003001: c.G149A, p.R50H (chr1: 161298257, rs769177037) in *SDHC* were also detected simultaneously in two patients (Pat102 and Pat152). Nine patients with CPGLs carried one pathogenic variant NM_003002: c.A305G, p.H102R (chr11: 111959726, rs104894302) in *SDHD*, including two patients (Pat1 and Pat5) with bilateral/multiple paragangliomas.

Pathogenic/likely pathogenic variants in *SDHB, SDHD*, and *RET* genes were found in 9 out of 23 (39%) patients with VPGLs (Supplementary Table 1, Table 2). The frequency of variants had the following distribution: *SDHB* (13%, 3/23), *SDHD* (22%, 5/23), and *RET* (4%, 1/23). All the patients with VPGLs carried pathogenic/likely pathogenic variants only in one of these genes. Notably, a likely pathogenic variant NM_020975: c.A2372T, p.Y791F (chr10: 43613908, rs77724903) in *RET* identified in four patients with CPGLs was also found in a patient with VPGL (Pat158). Moreover, three patients with VPGLs harbored the pathogenic variant NM_003002: c.A305G, p.H102R (chr11: 111959726, rs104894302) in the *SDHD* gene detected with high frequency in a set of CPGLs.

Next, we analyzed the age at diagnosis and sex ratio for *SDHB, SDHC, SDHD*, and *RET* variants within the cohort of patients with HNPGLs (Table 3). Variants in the *SDHC* and *SDHD* genes were diagnosed with approximately equal frequency in males and females taking into account 1:3 male to female ratio among the studied cohort. *SDHB* variants were found third-fold more frequent in females when *RET* variants were detected about two-fold more frequent in males. Variants in *SDHB, SDHC*, and *SDHD* were observed in all age groups and were more often detected in patients aged between 19–40 and 41–60 years. *RET* variants were identified in two groups of patients aged 19–40 and 41–60 years. Notably, frequency of variants in all the genes was the lowest in patients aged 61–80 years.

**Table 3:** Age and sex of patients with *SDHB, SDHC, SDHD*, and *RET* variants.

**Gene**	**Age at presentation**			**Sex (M:F)**
	**19–40 yr**	**41–60 yr**	**61–80 yr**	
***SDHB***	4	5	1	1:9
***SDHC***	2	2	1	1:4
***SDHD***	8	12	5	7:18
***RET***	4	1	0	2:4

*M, males; F, females*.

## Discussion

In HNPGLs, the occurrence and frequency of mutations in the *SDHx* genes are extensively studied and are of importance for the diagnosis and management of the disease. The prevalence of mutations in other susceptibility genes has been poorly investigated. Notably, most studies have been focused on germline variants. In this work, we cannot establish germline and somatic mutation status for identified variants, since we used the archival collection of tumors for which blood or other normal tissues were not available. We obtained data on the overall frequency of pathogenic/likely pathogenic variants in the main susceptibility genes that allows to better understand molecular basis of the tumor development in Russian patients. A more similar study was performed for 24 Spanish patients with HNPGLs, who were subjected to genetic testing for germline and somatic mutations in the *SDHx* genes (Curras-Freixes et al., 2015). In contrast to our data, the majority of mutations were detected in *SDHB* (33%, 8/24) followed by *SDHD* (21%, 5/24) and *SDHC* (4%, 1/24). Two patients with *SDHB* mutations and one patient with *SDHD* mutation were characterized by metastatic tumors but no *SDHA* variants were detected.

In Russian patients, we revealed the likely pathogenic variant in *RET* in 5% of cases (5/102), including four patients with CPGLs and one patient with VPGL. Activation mutations in the proto-oncogene *RET* lead to the development of an autosomal dominant syndrome called multiple endocrine neoplasia type 2 (MEN2). Up to 50% of patients with MEN2 develop PCCs (Pedulla et al., 2014). HNPGLs have been rarely described in patients with MEN2 syndrome (Boedeker et al., 2009). However, several studies have reported *RET* mutations in HNPGLs without any association with MEN2 syndrome. A *RET* mutation was previously detected in a patient with multiple paragangliomas (Ding et al., 2019). Moreover, the likely pathogenic germline variant NM_020975: c.A2372T, p.Y791F (chr10: 43613908, rs77724903) in *RET* was identified in two out of four members of a family with multiple and malignant paragangliomas (Choi Jdo et al., 2014). All four members carried pathogenic *SDHD* mutations. In addition, in this family, the *RET* mutation was observed in the male adult with bilateral carotid body and jugulotympanic paragangliomas and his son with unilateral CPGLs. Here, we also detected this *RET* variant in all *RET*-mutated tumors. Moreover, this variant co-occurred with the pathogenic start-loss variant in *SDHA* and the pathogenic splice site variant in *SDHB*. Collectively, data from our study and previous studies suggest that this *RET* variant can occur both alone and together with *SDHD, SDHA*, and *SDHB* pathogenic variants. However, according to the ClinVar database, this *RET* mutation was annotated as pathogenic variant associated with the MEN2 syndrome and its pathogenicity has not been proved by any functional studies. Thus, the role of the *RET* variant in the development of HNPGLs is controversial taking into account that we detected this variant in patients, who were not diagnosed with the MEN2 syndrome.

No pathogenic/likely pathogenic variants in *SDHAF2, VHL, MAX, MEN2, NF1, FH, TMEM127*, and *SLC25A11* were identified among the Russian patients. Variants in all the genes were previously reported only as germline at a very low frequency in HNPGLs (Boedeker et al., 2009; Burnichon et al., 2012; Castro-Vega et al., 2014; Bausch et al., 2017; Buffet et al., 2018; Guha et al., 2019).

In the study, we also analyzed male to female ratio and age groups for identified variants in the *SDHx* and *RET* genes. Currently, clinic-genetic correlations in association with mutations in these genes in HNPGLs have been poorly investigated. Moreover, reported data are related to germline mutations and can be compared only conditionally with our results. Thus, Mario Hermsen with colleagues studied 23 males and 51 females and with HNPGLs and found 8:6 and 5:9 ratios for *SDHB* and *SDHD* mutations, respectively (Hermsen et al., 2010). Taking into account the initial male to female ratio approximately 1:2, we can see that *SDHD* mutations were detected about equally in males and females that is concordance with our data. *SDHB* mutations were more frequently revealed in males compared with females, while we obtained opposite results. In addition, it was observed that *SDHB* and *SDHD* mutations were diagnosed predominantly in patients aged <50 years. We showed that patients aged <61 years carried variants in these genes more frequently. Also, age-related penetrance for *SDHB* and *SDHD* mutations was shown to be increased by 50 years in HNPGLs (Neumann et al., 2004). In our study, we also observed higher number of patients aged 41–60 years with *SDHB* and *SDHD* variants.

## Data Availability Statement

The datasets presented in this study can be found in online repositories. The names of the repository/repositories and accession number(s) can be found here: https://www.ncbi.nlm.nih.gov/bioproject/PRJNA639937.

## Ethics Statement

The studies involving human participants were reviewed and approved by Ethics Committee of Vishnevsky Institute of Surgery. The patients/participants provided their written informed consent to participate in this study.

## Author Contributions

AS and AVK: conceptualization and writing—review and editing. GK, VP, AD, AS, and NM: formal analysis. MF, EP, and ZG: investigation. GR, DK, and AG: resources. AP, GA, and ADK: writing—review and editing. All authors contributed to the article and approved the submitted version.

## Conflict of Interest

The authors declare that the research was conducted in the absence of any commercial or financial relationships that could be construed as a potential conflict of interest.
