# Supplementary material for: Mutation Frequency in Main Susceptibility Genes Among Patients With Head and Neck Paragangliomas
Source: Front Genet. 2020 Dec 18;11:614908. doi: 10.3389/fgene.2020.614908 (PMC7775293; doi:10.3389/fgene.2020.614908)
Supplement: Supplementary file 1 [file Data_Sheet_1.PDF]

Table 1. Pathogenic/likely pathogenic variants in the *SDHA*, *SDHB*, *SDHC*, *SDHD*, and *RET* genes in patients with HNPGLs.

**Pat**	**Tumor studied**	**Sex**	**Age**	**Gene**	**Pos**	**Mut**	**Change**	**dbSNP ID**	**ClinSig**
Pat16[a]	CPGLs	F	29	*RET*	chr10: 43613908	Missense	NM_020975: c.A2372T/p.Y791F	rs77724903	LP[†]
Pat35	CPGLs	F	30						
Pat142[a]	CPGLs	M	40						
Pat155	CPGLs	F	58						
Pat158	VPGLs	F	34						
Pat16[a]	CPGLs	F	29	*SDHA*	chr5: 218471	Start-lost	NM_004168.3: c.A1G/p.M1?	rs1061517;239661	P[††]
Pat6	CPGLs	F	39	*SDHB*	chr1: 17359564	Missense	NM_003000.2: c.T277C/ p.C93R	rs727503415;165180	LP[†]
Pat28	VPGLs	F	26	*SDHB*	chr1:	Stop-gain	NM_003000: c.C79T/p.R27X	rs74315369	P[††]

					17371377				
Pat11	CPGLs	F	70	*SDHB*	chr1: 17349105	Stop-gain	NM_003000: c.A763T/ p.K255*	-	P†
Pat46	CPGLs	F	49	*SDHB*	chr1: 17349144	Missense	NM_003000: c.C724T/p.R242C	rs786203251	P††
Pat40	VPGLs	F	56	*SDHB*	chr1: 17350571	Splice site	NM_003000: c.541-2A>G	rs786201161	P††
Pat47*	CPGLs	F	22	*SDHB*	chr1: 17355175	Stop-gain	NM_003000.2: c.C343T/p.R115*	rs751000085;197210	P††
Pat101*	CPGLs	F	46	*SDHB*	chr1: 17371320	Stop-gain	NM_003000.2: c.C136T/p.R46*	rs74315370	P††
Pat125	VPGLs	F	47	*SDHB*	chr1: 17355233	Splice site	NM_003000: c.287-2A>G	rs1064794270	P††
Pat112	CPGLs	F	47	*SDHB*	chr1:	Missense	NM_003000: c.G725A/p.R242H	rs74315368	P††

					17349143				
Pat142[a]	CPGLs	M	40	*SDHB*	chr1: 17359554	Splice site	NM_003000: c.286+1G>A	rs786201063	P[††]
Pat10	CPGLs	F	35	*SDHC*	chr1: 161310387	Stop-gain	NM_003001: c.G183A/p.W61*	-	P[†]
Pat27	CPGLs	F	54	*SDHC*	chr1: 161332121	Frameshift	NM_003001: c.409delT/p.W137fs	-	P[†]
Pat41	CPGLs	F	52	*SDHC*	chr1: 161310428	Missense	NM_003001: c.G224A/p.G75D	rs786205147	LP[†]
Pat102	CPGLs	F	31	*SDHC*	chr1: 161298257	Missense	NM_003001: c.G149A/p.R50H	rs769177037	LP[†]
Pat152*	CPGLs	M	63						
Pat1[#b]	CPGLs and VPGLs	F	58	*SDHD*	chr11: 111959726	Missense	NM_003002: c.A305G/p.H102R	rs104894302	P[†]

Pat5#	CPGLs	F	61						
Pat22	CPGLs	F	63						
Pat53	CPGLs	M	48						
Pat56	CPGLs	F	50						
Pat67	CPGLs	F	55						
Pat100	CPGLs	F	35						
Pat113	CPGLs	F	55						
Pat143	CPGLs	F	38						
Pat68	VPGLs	F	68						
Pat120	VPGLs	F	56						
Pat2	CPGLs	F	33	*SDHD*	chr11: 111958640	Stop-gain	NM_003002: c.C112T/p.R38*	rs80338843	P††
Pat38#	CPGLs	F	39						

Pat107	CPGLs	M	61						
Pat3	CPGLs	F	34	*SDHD*	chr11: 111957643	Frameshift	NM_003002: c.13dupT/p.W5fs	-	P†
Pat7	CPGLs	F	54	*SDHD*	chr11: 111957632	Missense	NM_003002: c.A1T/p.M1L	rs104894307	P††
Pat8	CPGLs	M	22	*SDHD*	chr11: 111959631	Missense	NM_003002: c.G210C/p.R70S	-	LP†
Pat21	CPGLs	M	63	*SDHD*	chr11: 111959637	Frameshift	NM_003002: c.217dupA/p.S73fs	-	LP†
Pat35	CPGLs	F	30	*SDHD*	chr11: 111959626	Stop-gain	NM_003002: c.G205T/p.E69*	-	P†
Pat128#b	CPGLs and VPGLs	M	54						
Pat36	CPGLs	M	59	*SDHD*	chr11: 111959639	Frameshift	NM_003002: c.220_228delGTTTTGCTCinsT/p.V74fs	-	LP†

Pat55[#b]	CPGLs and VPGLs	M	54	*SDHD*	chr11: 111965547	Frameshift	NM_003002: c.334_337del/p.T112fs	rs587776648	P[††]
Pat62	CPGLs	F	57	*SDHD*	chr11: 111959715	Frameshift	NM_003002: c.295_298del/p.L99fs	rs786203067	LP[†]
Pat110*	CPGLs	F	46	*SDHD*	chr11: 111965566	Frameshift	NM_003002: c.353delA/p.D118fs	-	P[†]
Pat149	CPGLs	F	19	*SDHD*	chr11: 111965567	Frameshift	NM_003002: c.354_355del/p.D118fs	-	LP[†]

Pat, patient; Pos, position; Mut, type of mutations; ClinSig, clinical significance; F, female; M, male; P, pathogenic; LP, likely pathogenic; [†]ACMG-AMP interpretation of pathogenicity; [††]ClinVar interpretation of pathogenicity; *patients with the tumor recurrence; [#]patients with bilateral/multiple PGLs; [a]patients with more than one pathogenic/likely pathogenic variant; [b]patients with CPGLs and VPGLs studied in both sample sets simultaneously.
